# AhrC Negatively Regulates Streptococcus mutans Arginine Biosynthesis

**DOI:** 10.1128/spectrum.00721-22

**Published:** 2022-08-08

**Authors:** Meiling Jing, Ting Zheng, Tao Gong, Jiangchuan Yan, Jiamin Chen, Yongwang Lin, Boyu Tang, Qizhao Ma, Xuedong Zhou, Yuqing Li

**Affiliations:** a State Key Laboratory of Oral Diseases, National Clinical Research Center for Oral Diseases, West China Hospital of Stomatology, Sichuan University, Chengdu, China; b Department of Operative Dentistry and Endodontics, West China Hospital of Stomatology, Sichuan University, Chengdu, China; c Department of Pediatric Dentistry, West China Hospital of Stomatology, Sichuan University, Chengdu, China; McGill University

**Keywords:** biofilm(s), *Streptococcus mutans*, transcription factor(s), gene expression, microbial genetics

## Abstract

Streptococcus mutans is a primary cariogenic pathogen in humans. Arginine metabolism is required for bacterial growth. In S. mutans, however, the involvement of transcription factors in regulating arginine metabolism is unclear. The purpose of this study was to investigate the function and mechanism of ArgR family transcription factors in S. mutans. Here, we identified an ArgR (arginine repressor) family transcription factor named AhrC, which negatively regulates arginine biosynthesis and biofilm formation in S. mutans. The *ahrC* in-frame deletion strain exhibited slow growth and significantly increased intracellular arginine content. The strain overexpressing *ahrC* showed reduced intracellular arginine content, decreased biofilm biomass, reduced production of water-insoluble exopolysaccharides (EPS), and different biofilm structures. Furthermore, global gene expression profiles revealed differential expression levels of 233 genes in the *ahrC*-deficient strain, among which genes related to arginine biosynthesis (*argJ*, *argB*, *argC*, *argD*, *argF*, *argG*, *argH*) were significantly upregulated. In the *ahrC* overexpression strain, there are 89 differentially expressed genes, mostly related to arginine biosynthesis. The conserved DNA patterns bound by AhrC were identified by electrophoretic mobility shift assay (EMSA) and DNase I footprinting. In addition, the analysis of β-galactosidase activity showed that AhrC acted as a negative regulator. Taken together, our findings suggest that AhrC is an important transcription factor that regulates arginine biosynthesis gene expression and biofilm formation in S. mutans. These findings add new aspects to the complexity of regulating the expression of genes involved in arginine biosynthesis and biofilm formation in S. mutans.

**IMPORTANCE** Arginine metabolism is essential for bacterial growth. The regulation of intracellular arginine metabolism in Streptococcus mutans, one of the major pathogens of dental caries, is unclear. In this study, we found that the transcription factor AhrC can directly and negatively regulate the expression of *N*-acetyl-gamma-glutamyl-phosphate reductase (*argC*), thus regulating arginine biosynthesis in S. mutans. In addition, the *ahrC* overexpression strain exhibited a significant decrease in biofilm and water-insoluble extracellular polysaccharides (EPS). This study adds new support to our understanding of the regulation of intracellular arginine metabolism in S. mutans.

## INTRODUCTION

Dental caries is one of the most common diseases in the world and brings a serious burden to health and economic factors (1). It is a multifactorial disease where the accumulation of plaque biofilm is an important contributing factor (2, 3). Streptococcus mutans is now referred to as a keystone pathogen that has various caries-causing virulence factors, including acid production, acid-resistance, extracellular polysaccharide (EPS) synthesis, adhesion ability to the dental surface, and strong environmental adaptability (4). Targeted inhibition of S. mutans is an important research direction for caries prevention and treatment.

Arginine is considered to be a promising agent for caries management (5). Arginine can be metabolized by arginine deiminase system (ADS)-positive bacteria (including Streptococcus sanguinis, Streptococcus gordonii, Streptococcus parasanguinis, Streptococcus mitis, certain lactobacilli and *Actinomyces* strains) to form ammonia and ATP, which can raise the pH in the cytoplasm and environment and provide energy (5, 6). In addition, arginine can reduce S. mutans growth, biofilm formation, EPS synthesis, acid production, and tolerance of environmental stress (7, 8). Besides, arginine can be used as a precursor for protein synthesis and an important target for posttranslational modifications of proteins, which is involved in the basic life activities of bacteria (9, 10).

The ArgR family transcriptional regulator is one of the DNA-binding transcription factors, with the most common function being to regulate arginine metabolism and transport (11, 12). Meanwhile, the DNA-binding domain that defines the ArgR family is the “arginine repressor C-terminal domain” (13, 14). In addition, the ArgR family is considered to be related to modulating bacterial resilience under various environmental stresses, such as elevated osmotic pressure, low pH, and oxidative stresses (15–17). The ArgR family transcription regulators ArgR1 and AhrC were reported to regulate the expression of *aliB* (encoding an oligopeptide-binding lipoprotein), which is important for nasopharyngeal colonization in the human pathogen Streptococcus pneumoniae (18). In Pseudomonas protegens Pf-5, ArgR inhibits lipase gene *lipA* at the transcriptional level by directly binding to the *lipA* promoter (19). The ArgR family transcription factor AhrC is reported to activate the expression of adhesive pili and the collagen-binding adhesin Ace in Enterococcus faecalis (20).

Additionally, the ArgR family has a role in biofilm formation in S. gordonii (21), E. faecalis (22), and S. sanguinis SK36 (23). In Pseudomonas aeruginosa, the two-component regulatory system NarX-NarL regulates anaerobic metabolism by inhibiting ArgR-mediated arginine-dependent activation of the *arcDABC* operon, which is important for biofilm growth (24, 25). Recent research showed that a new coumarin compound, 3,3′-(3,4-dichlorobenzylidene)-bis-(4-hydroxycoumarin) termed DCH, reduced biofilm formation by affecting the arginine catabolic pathway through binding to ArgR in methicillin-resistant Staphylococcus aureus (26).

However, the role of ArgR family transcriptional regulators in S. mutans is not clear. According to NCBI annotation, we identified the ArgR family transcriptional regulator (AhrC; SMU.584), which had 57% similarity to AhrC in E. faecalis. Interestingly, the order of genes around *ahrC* is similar in S. mutans and E. faecalis (see Fig. S1 in the supplemental material). Here, the purpose of this study was to investigate the function and mechanism of the ArgR family transcription factor AhrC in S. mutans. The data in this study showed that AhrC regulates arginine synthesis and biofilm formation in S. mutans. We also found that AhrC directly binds to the promoter of *argC*, negatively regulating arginine biosynthesis gene expression. These findings add new aspects to the regulation of genes involved in arginine metabolism by S. mutans and provide a potential target for managing dental caries.

## RESULTS

### Deletion of *ahrC* affected the growth characteristics and intracellular arginine content of S. mutans.

According to the National Center for Biotechnology Information (NCBI), *ahrC* encodes a putative ArgR family transcriptional regulator whose function has yet to be elucidated. A markerless in-frame deletion mutant (Δ*ahrC* mutant) and an overexpression strain (UA159/pDL278-*ahrC*) for *ahrC* were constructed, confirmed, and characterized to gain insight into the function of *ahrC* in S. mutans. For comparison, the *ahrC* deletion mutant complement was constructed with an *ldh* promoter (Δ*ahrC*/pDL278-*ahrC*), and UA159/pDL278 was constructed as a control for UA159/pDL278-*ahrC*.

**FIG 1:**
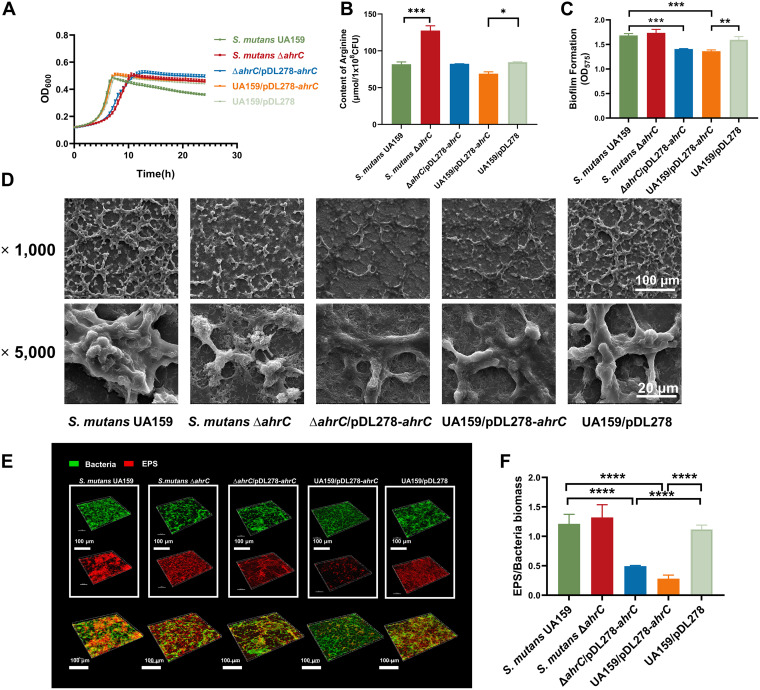
The growth characteristics, the content of arginine, and biofilm formation of S. mutans. (A) Growth curves of S. mutans strains grown in BHI medium for 24 h. (B) The content of arginine in S. mutans strains. (C) The biofilm biomass of S. mutans strains grown in BHIS medium for 24 h. (D) The SEM images show the 6-h biofilms of S. mutans strains. Images were taken at ×1,000 and ×5,000 magnification. (E) Double-labeled images of 6-h S. mutans strain biofilms. Red represents the EPS (Alexa Flour 647), and green represents the bacteria (SYTO 9). Images were taken at ×60 oil magnification and analyzed with IMARIS 9.0. Representative images are shown from at least five randomly selected fields of each sample. (F) The EPS/bacteria ratio of 6-h S. mutans biofilms. The data were quantified with ImageJ. The results were the mean values of five randomly selected areas in each sample, expressed as mean ± standard deviation.

The growth curve of these strains was measured to evaluate the effects of *ahrC* on the growth of S. mutans. UA159 appeared to have a swifter loss of viability following attainment of peak growth, while the other strains did not. The slopes during the exponential growth phase do not appear to be similar for all strains. A significant lag in growth and shallower slopes during the exponential growth phase were observed in the brain heart infusion (BHI) medium for the Δ*ahrC* and Δ*ahrC*/pDL278-*ahrC* strains compared to those in UA159. UA159/pDL278-*ahrC* and UA159/pDL278 exhibited no significant change in growth rate compared to that of UA159 (Fig. 1A).

Liquid chromatography-mass spectrometry (LC-MS) showed that the intracellular arginine content in the Δ*ahrC* strain was significantly upregulated; however, it decreased in the overexpressed strain (Fig. 1B).

These findings demonstrated a close relationship between *ahrC* and the growth characteristics and intracellular arginine content of S. mutans.

### Deletion and overexpression of *ahrC* impacted EPS-matrix assembly and altered three-dimensional architecture of S. mutans biofilm.

Since biofilm formation is a crucial driver of S. mutans pathogenicity, we were interested in determining whether *ahrC* affected the ability to form biofilms or not. Therefore, the biofilm biomass of the strains was compared through crystal violet staining. The UA159/pDL278-*ahrC* and Δ*ahrC*/pDL278-*ahrC* strains exhibited the decreased biomass of S. mutans biofilms, while the Δ*ahrC* strain showed no significant change (Fig. 1C).

The morphology of the S. mutans biofilms was further assessed at early (6 h), middle (12 h), and mature (24 h) phases by scanning electron microscopy (SEM). The biofilms formed by UA159 have a three-dimensional (3D) appearance, with bacterial cells clustered in clumps and connected by many crisscrossing strips of extracellular polymer substance that embedded the bacterial cells. The UA159 biofilms showed a more stereoscopic appearance and more extracellular matrix at later phases than early phases. In contrast, the Δ*ahrC* strain formed a smoother structure with fewer isolated bacterial cell islets and less extracellular polymer substance. Also, the UA159/pDL278-*ahrC* strain formed a flatter structure with less extracellular matrix at three phases (Fig. 1D; see also Fig. S2 in the supplemental material).

A confocal laser scanning microscope (CLSM) was used to analyze the biofilm (Fig. 1E). With bacteria in green and EPS in red, typical pictures of 3D renderings of the EPS-microcolony complex are presented. At the early phase, fewer bacteria and EPS were formed in the biofilm by the UA159/pDL278-*ahrC* strain; meanwhile, the EPS decreased more significantly. There is a significant decrease in biofilm biomass of the Δ*ahrC*, Δ*ahrC*/pDL278-*ahrC*, and UA159/pDL278-*ahrC* strains (see Fig. S3A in the supplemental material). Meanwhile, the biofilm thickness of the Δ*ahrC*/pDL278-*ahrC* and UA159/pDL278-*ahrC* strains also showed a significant decrease (Fig. S3B). The EPS/bacteria ratio was calculated to further ensure that the UA159/pDL278-*ahrC* strain had fewer EPS than the wild type (Fig. 1F). However, the Δ*ahrC*/pDL278-*ahrC* strain also showed a decrease in EPS.

We noticed that the biofilm of the Δ*ahrC*/pDL278-*ahrC* strain was more similar to that of UA159/pDL278-*ahrC*. Thus, we measured the gene expression of *ahrC* in the Δ*ahrC*/pDL278-*ahrC* and UA159/pDL278-*ahrC* strains by quantitative PCR assay. The results showed that the expression level of *ahrC* in the Δ*ahrC*/pDL278-*ahrC* strain is similar to that in the UA159/pDL278-*ahrC* strain and significantly higher than that in UA159 (Fig. S3C). The similarity in *ahrC* expression is probably the reason why the complemented strain did not restore the phenotype.

These findings indicated that *ahrC* affected the architecture of biofilms, and the UA159/pDL278-*ahrC* strain biofilm contained fewer EPS than the UA159 strain.

### Transcriptome analysis of the Δ*ahrC* and UA159/pDL278-*ahrC* strains.

Since we found that *ahrC* significantly affected the arginine production and biofilm formation of S. mutans, the transcriptome of the Δ*ahrC* and UA159/pDL278-*ahrC* strains was analyzed to identify changes in whole-gene expression.

**FIG 2:**
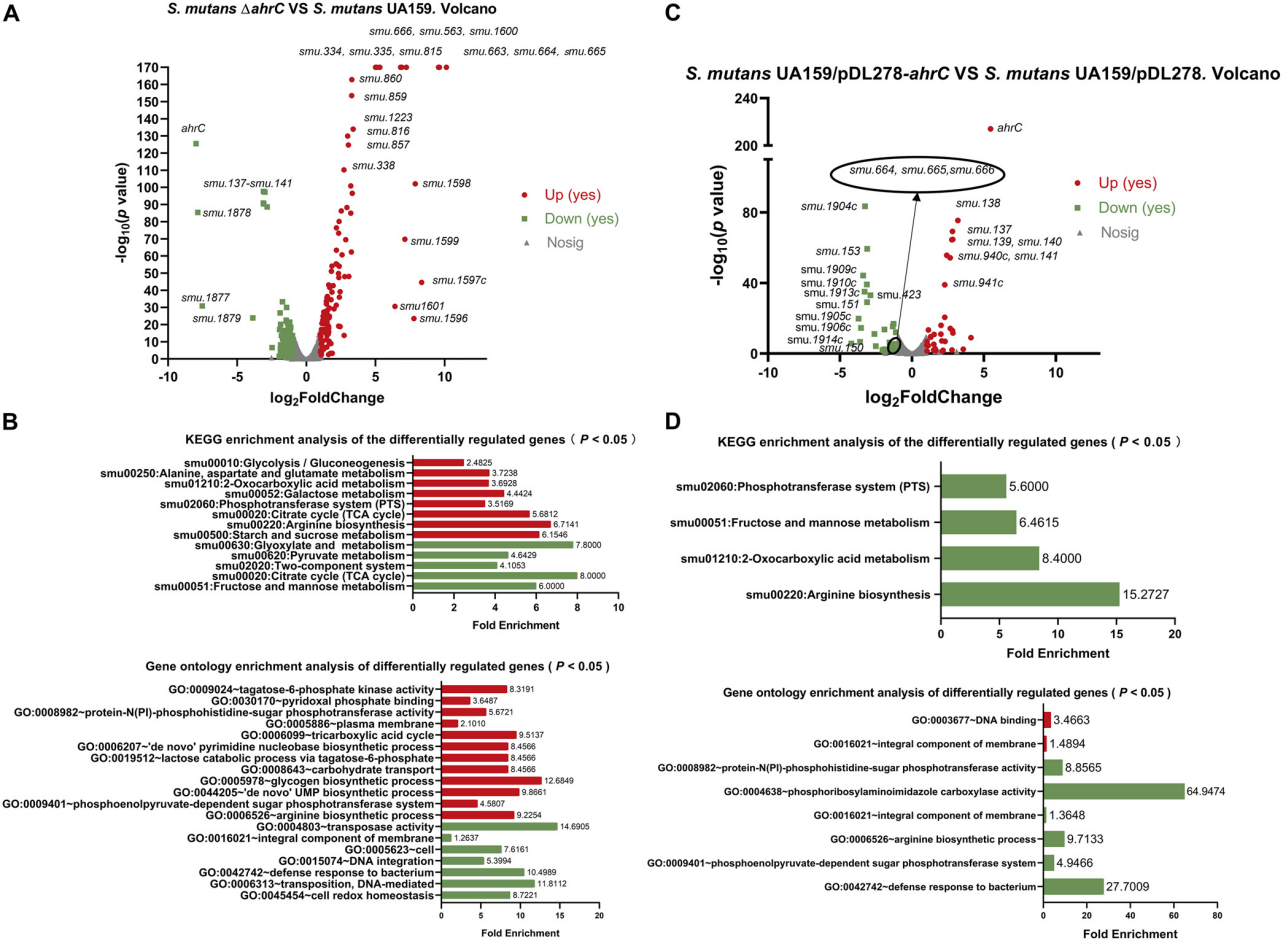
Transcriptomic analysis of the Δ*ahrC* and UA159/pDL278-*ahrC* strains. (A) Volcano plot showing the differences in gene expression between S. mutans UA159 and the Δ*ahrC* strain. (B) The KEGG pathway analysis and the GO enrichment analysis of DEGs in the Δ*ahrC* strain. (C) Volcano plot showing the gene expression differences between S. mutans UA159/pDL278 and S. mutans UA159/pDL278-*ahrC*. (D) The KEGG pathway analysis and the GO enrichment analysis of DEGs in S. mutans UA159/pDL278-*ahrC*. Upregulated genes are shown in red, whereas downregulated genes are shown in green. GO, gene ontology; KEGG, Kyoto Encyclopedia of Genes and Genomes.

**FIG 3:**
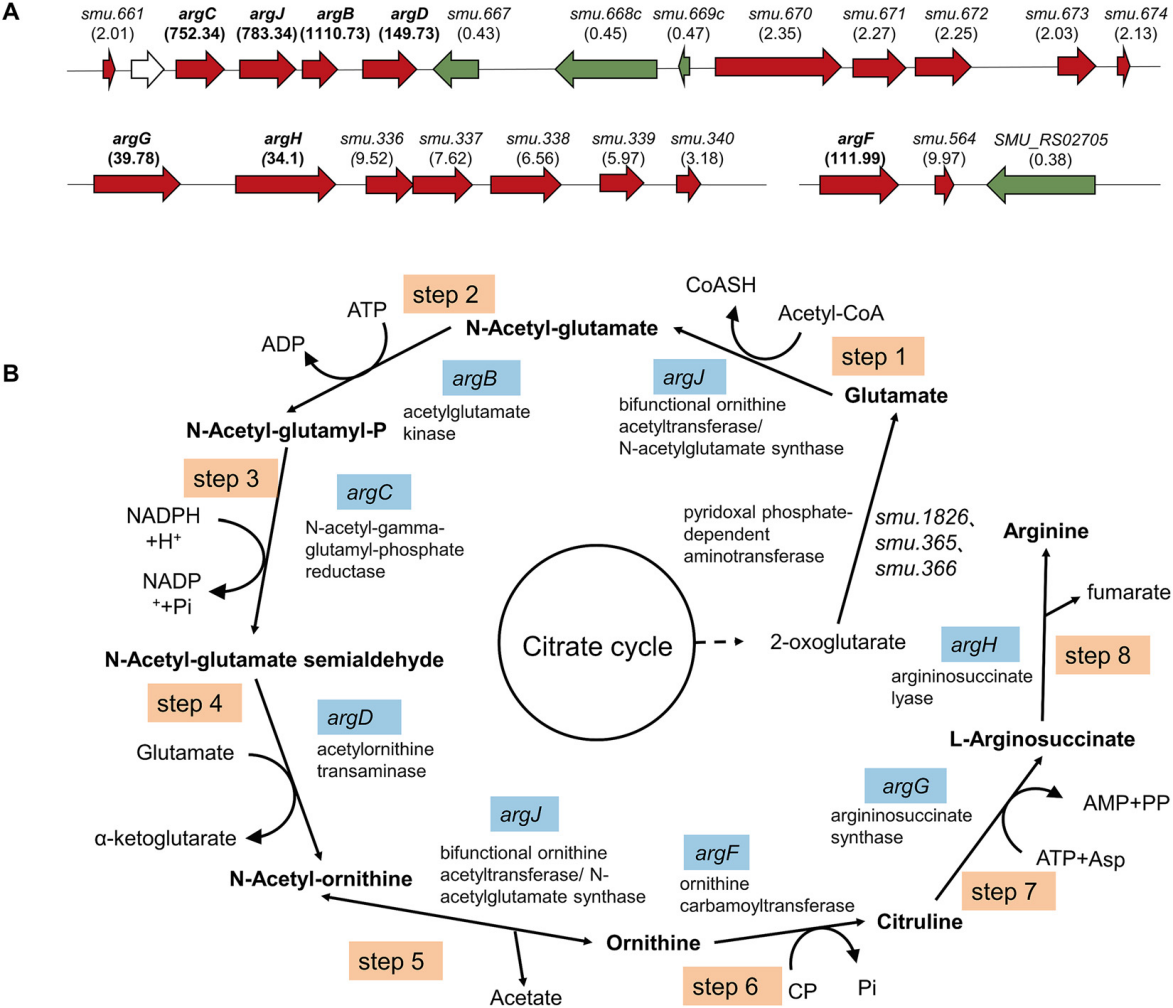
Genes regulating the arginine biosynthesis pathway. (A) The differentially expressed arginine biosynthesis operons in the S. mutans Δ*ahrC* strain. The genetic organization of differentially expressed gene clusters associated with arginine biosynthesis in the S. mutans Δ*ahrC* strain. Upregulated genes are indicated in red, whereas downregulated genes are shown in green, with the fold of differential expression represented by the values in parentheses. (B) Flowchart of the arginine biosynthesis pathway in S. mutans. Genes encoding enzymes in the arginine synthesis pathway are indicated in blue.

In the Δ*ahrC* strain, 131 significantly upregulated and 102 downregulated genes were identified compared to UA159 (Fig. 2A; see also Table S3 in the supplemental material). According to the S. mutans UA159 genome annotation obtained from the NCBI, the most obviously upregulated genes with known functions, including *smu.334* (*argG*), *smu.335* (*argH*), *smu.563* (*argF*), and *smu.663* to *smu.666* (*argC*, *argJ*, *argB*, and *argD*) were mainly associated with arginine biosynthesis (Fig. 3A and B; see also Table S3). In addition, *ptsH* (encoded the heat-stable phosphocarrier protein HPr), the cellobiose-specific phosphotransferase system (PTS) operon (*celA*, *celB*, *celC*, *celD*, *smu.1597c*, and *celR*), the sorbitol-PTS operon (*smu.311*, *smu.312*, and *smu.313*), and the lactose-PTS operon (*lacABCDEF*) were also upregulated (27–29). The genes associated with pyrimidine metabolism, including *smu.856* to *smu.860* and *smu.1223*, were upregulated. The putative amino acid transporters *smu.815* and *smu.817* and the aminotransferase *smu.816* were all upregulated. The mannose-PTS operon (*manLMN*) and the fructose-PTS operon (*fruKRI*) were downregulated. The genes related to malolactic fermentation (*smu.137* to *smu.139*) were also significantly downregulated in the Δ*ahrC* strain (30).

We further conducted gene enrichment and functional annotation clustering analysis of differentially expressed genes (DEGs). In the Δ*ahrC* strain, DEGs were enriched in 13 Kyoto Encyclopedia of Genes and Genomes (KEGG) pathways (Fig. 2B). The DEGs were mainly involved in arginine biosynthesis; starch and sucrose metabolism; citrate cycle (tricarboxylic acid [TCA] cycle); glyoxylate and metabolism; PTS; galactose metabolism; 2-oxocarboxylic acid metabolism; fructose and mannose metabolism; alanine, aspartate, and glutamate metabolism; glycolysis/gluconeogenesis; and pyruvate metabolism. The gene ontology (GO) term enrichment analysis revealed that DEGs in the Δ*ahrC* strain primarily belonged to the arginine biosynthetic process, tagatose-6-phosphate kinase activity, protein-N(PI)-phosphohistidine-sugar phosphotransferase activity, tricarboxylic acid cycle, *de novo* pyrimidine nucleobase biosynthetic process, lactose catabolic process via tagatose-6-phosphate, carbohydrate transport, glycogen biosynthetic process, *de novo* UMP biosynthetic process, and phosphoenolpyruvate-dependent sugar phosphotransferase system.

Thirty-four significantly upregulated and 55 downregulated genes were identified in the UA159/pDL278-*ahrC* strain compared to those in UA159/pDL278 (Fig. 2C; see also Table S4 in the supplemental material). According to the S. mutans UA159 genome annotation obtained from the NCBI, the downregulated genes with known functions were mainly associated with arginine biosynthesis and carbohydrate transport and metabolism. Furthermore, part of TnSmu1, including *smu.191c* to *smu.214c*, was upregulated in the UA159/pDL278-*ahrC* strain (Fig. 2C; see also Table S3). In addition, the gene cluster related to malolactic fermentation (s*mu.137* to *smu.139*) and the gene related to biofilm formation (*smu.940c*) were significantly upregulated in UA159/pDL278-*ahrC* (31).

DEGs in UA159/pDL278-*ahrC* were mainly involved in the phosphotransferase system (PTS), fructose and mannose metabolism, 2-oxocarboxylic acid metabolism, and arginine biosynthesis (Fig. 2D). In addition, the GO enrichment analysis showed that DEGs were involved in the protein-N(PI)-phosphohistidine-sugar phosphotransferase activity, phosphoribosylaminoimidazole carboxylase activity, arginine biosynthetic process, and phosphoenolpyruvate-dependent sugar phosphotransferase system.

In summary, the S. mutans gene *ahrC* is mainly associated with arginine biosynthesis, carbohydrate transport and catabolism, glycogen biosynthesis, protein transport and metabolism, and nucleotide biosynthesis.

### AhrC directly bound to the promoter of arginine biosynthesis gene cluster.

**FIG 4:**
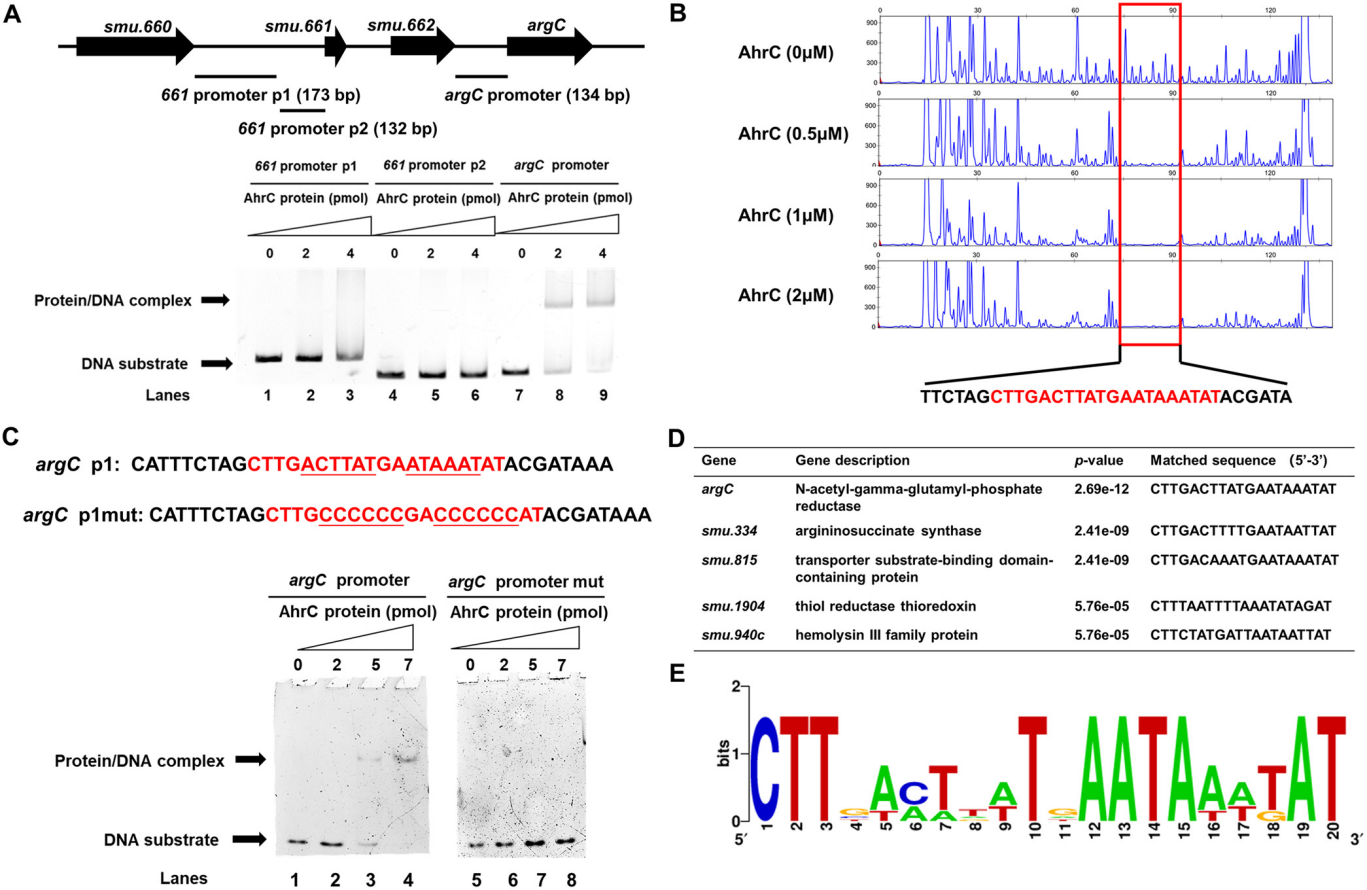
Identification of conserved DNA motif bound by AhrC. (A) The result of electrophoretic mobility shift assay (EMSA) showing AhrC protein binding to the *argC* promoter (lanes 7 to 9). (B) DNase I footprinting experiments showing the protective effect of AhrC protein (0, 0.5, 1, and 2 μM) against 5-FAM-labeled primers of *argC* promoter. The sequence of the protected area is indicated in red. (C) The conserved palindromic sequences and mutated sequences of *argC* promoter. DNA binding activity of AhrC on *argC* promoter p1 (lanes 1 to 4) and *argC* promoter p1mut (lanes 5 to 8). (D) The prediction of AhrC binding sequence in selected gene promoters. (E) The sequence logo for AhrC binding motif was generated by the WebLogo tool.

The gene *argC* is in an upstream position of the arginine biosynthesis gene cluster and increased by about 752 fold in the Δ*ahrC* strain (Fig. 3A; see also Table S3). As transcription factors usually bind to gene promoters to control their expression, the promoter region of *argC* was used to study the DNA binding activity and specificity of AhrC through electrophoretic mobility shift assay (EMSA). The promoters of *ahrC* and *smu.661* were used as negative controls for EMSA. When AhrC was incubated with *argC* promoter, changes were observed in mobility. No protein/DNA complex was detected after incubation with *smu.661* promoter and *ahrC* promoter (Fig. 4A; see also Fig. S4 in the supplemental material), indicating that AhrC could specifically bind to the *argC* promoter region.

DNase I footprinting experiments were further used to show the protective effect of AhrC protein against 5-FAM (5-carboxyfluorescein)-labeled primers of the *argC* promoter. As the protein concentration increased, the DNA region 5′-CTTGACTTATGAATAAATAT-3′ was protected by AhrC, a short 20-bp palindrome. To further ensure the specificity of the DNA motif for AhrC, *argC* p1 and p1mut were designed. The two DNA fragments contained the 20-bp palindrome while p1mut exhibited a change of the key A/T bases to G/C, and *argC* p1 could bind to the AhrC while p1mut could not form the protein/DNA complex (Fig. 4C), indicating that DNA motif 5′-CTTGACTTATGAATAAATAT-3′ is the binding site identified by AhrC. The AhrC-binding sequences inferred from the selected gene promoters were predicted by MEME tools (https://meme-suite.org/meme/tools/meme) (Fig. 4D) (32). The inferred AhrC-binding sequences were found in the *argG*, *smu.815*, *smu.1904*, and *smu.940c* promoter region. The AhrC-binding sequences inferred by MEME were visualized using WebLogo with a view to finding a conserved sequence (33) (Fig. 4E).

### AhrC served as a negative regulator.

**FIG 5:**
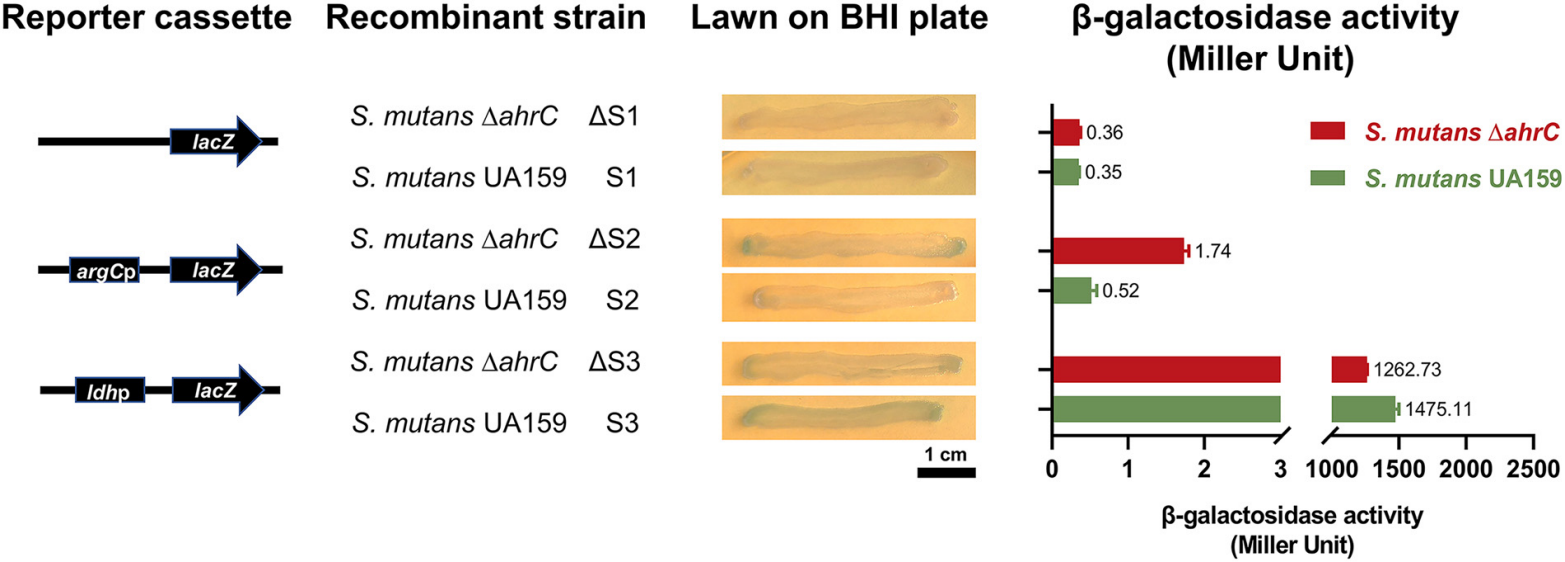
AhrC served as a suppressor. Constructing a series of *lacZ* and promoter-*lacZ* coexpression plasmids to analyze the effect of AhrC on *argC* expression. A schematic representation of each cloned reporter box is used to produce a recombinant strain. The *lacZ* and *ldh*p-*lacZ* plasmids were used as controls. The recombinant strains were inoculated on BHI-agar plates containing 1 mg/mL spectinomycin and 40 μg/mL X-gal for 48 h of incubation. The results of β-galactosidase activity analysis were expressed in Miller units. The data consisted of three replicates, expressed as mean ± standard deviation.

A series of promoter *lacZ* reporter plasmids were constructed using β-galactosidase as the reporter gene to figure out the function of AhrC on *argC* expression. The *ldh* promoter (positive control) significantly promoted *lacZ* expression (Fig. 5). The corresponding UA159 and Δ*ahrC* strains showed blue and higher β-galactosidase activity than nonpromoter *lacZ* plasmids. With an *argC* promoter, the Δ*ahrC* strain was remarkably bluer, with higher β-galactosidase activity than UA159 (Fig. 5). Taken together, the present study data suggest that AhrC, acting as a negative regulator, binds to the *argC* promoter.

## DISCUSSION

In prokaryotic cells, the biosynthesis of arginine involves eight consecutive enzymes (34). According to the KEGG, the first and fifth steps of S. mutans arginine biosynthesis are catalyzed by bifunctional ornithine acetyltransferase (*argJ*), with a role similar to *N*-acetylglutamate synthase (Fig. 3B). It is reported that the bifunctional *argJ* has been found in Aspergillus fumigatus, Saccharomyces cerevisiae, and Thermophilic microorganisms (35–37). The present study showed that *ahrC* deletion significantly upregulated *argB*, *argC*, *argD*, *argF*, *argJ*, *argG*, and *argH*, indicating that *ahrC* is closely associated with the S. mutans arginine biosynthesis pathway. Moreover, the LC-MS results showed a significant increase in arginine content in the Δ*ahrC* strain and a significant decrease in arginine content in UA159/pDL278-*ahrC*, further indicating that *ahrC* regulates arginine biosynthesis in S. mutans.

The ability of S. mutans to form biofilms and produce EPS is critical for its pathogenicity and the development of dental caries. In this study, biofilm formation and EPS production decreased substantially and significantly in UA159/pDL278-*ahrC* compared to that in UA159 and UA159/pDL278, while the expression of *gtf*s, which encodes the major EPS synthesis enzymes in S. mutans, did not change appreciably. Further investigation is needed to find out whether the translation or export of the Gtfs is affected in UA159/pDL278-*ahrC*.

In addition, many recent studies have identified a relationship between an amino acid deficiency and biofilm formation (38–39). It was reported that methionine deficiency in staphylococci and P. aeruginosa significantly impaired their ability to form and maintain biofilms (38). A previous study showed that increased intracellular l-tryptophan concentration in E. coli triggered the degradation of their mature biofilms and decreased the intracellular l-tryptophan concentration induced biofilm formation increase (39). Besides, the Pseudomonas biofilm can perceive nutrient availability and readapts its metabolism to tune its own formation and dispersion in turn (40). In the present study, biofilm formation and EPS production decreased in S. mutans when intracellular arginine content was reduced, suggesting that intracellular arginine deficiency in S. mutans may affect its biofilm formation and EPS production.

Indeed, there have been many studies demonstrating the effect of exogenous l-arginine on biofilm formation and acid production of oral bacteria (41–43). However, studies on the relationship between arginine biosynthesis and biofilm formation are relatively scarce. In S. sanguinis SK36, CiaR promotes biofilm formation by suppressing *argB* transcription, and the deletion of *argR*, *argC*, *argG*, *argH*, and *argJ* also promotes biofilm formation (23). In S. gordonii, ArgR family transcription factor ArcR acts as a key determinant of biofilm formation without affecting arginine metabolic gene expression (21). In Pseudomonas putida, both exogenous and endogenous arginine influence biofilm formation and alter extracellular matrix through changes in cyclic diguanylate (44). In this study, we determined that AhrC is a key factor in biofilm formation and affects arginine biosynthesis, suggesting a close correlation between arginine biosynthesis and biofilm formation in S. mutans.

The PTS system is required for sugar transport and metabolism, affecting biofilm formation and bacterial virulence (45). For instance, it is well known that the glucose-mannose-PTS, especially *manL*, participates in carbon catabolite repression (CCR; 46, 47). Energy availability sensing through PTS is very important for S. mutans biofilms (48). In this study, we found that the knockdown of *ahrC* resulted in a significant decrease in the expression of *manLMN* and *fruRKI*. Overexpression of *ahrC* resulted in downregulation of *fruPC* and *levDEFG* operon expression. As a result, we hypothesized that *ahrC* might play a key role in the regulation of carbohydrate transport and metabolism as well as in CCR control.

According to NCBI annotation, *ahrC* is part of an operon associated with DNase and recombination activities. However, the expression of the operon genes (*smu.580* to *smu.583* and *smu.585*) was not altered in the Δ*ahrC* and UA159/pDL278-*ahrC* strains. Additionally, it was found in S. mutans that deletion of *immA*, the gene encoding the bacteriocin immunity protein, retarded growth in the early stages, reduced antimicrobial susceptibility, reduced biofilm thickness, and decreased the number of viable bacteria in the intermedium layer of the membrane (49). In addition, it has been reported that the *copYAZ* operon in S. mutans is involved in regulating copper efflux, biofilm formation, genetic transformation (50). In UA159/pDL278-*ahrC*, *immA* and *copYA* expression decreased significantly, contributing to its reduced biofilm and reduced EPS synthesis.

In the Δ*ahrC* strain, the expression of genes related to oxidative stress tolerance, such as *trxB* (encoding thioredoxin reductase), *sod* (encoding superoxide dismutase), *ahpC*/*F* (encoding alkyl hydroperoxide reductase C and F subunits), and *tpx* (encoding thiol peroxidase), were significantly decreased. However, we did not observe significant differences in the oxidative stress tolerance phenotype. The relationship between AhrC and oxidative stress tolerance requires further investigation.

In summary, in this study, we identified an ArgR family transcription factor AhrC that negatively regulates arginine biosynthesis in S. mutans. When the biosynthesis of endogenous arginine was decreased, biofilm formation and EPS production in S. mutans decreased significantly, suggesting that this biosynthetic pathway is a promising target for caries prevention.

## MATERIALS AND METHODS

### Bacterial strains and growth conditions.

Tables S1 and S2 in the supplemental material list all of the bacterial strains, plasmids, and primers used in this study. S. mutans UA159 and its derivatives were routinely grown in brain heart infusion (BHI) broth (Difco, Sparks, MD, United States) or on BHI agar at 37°C in an anaerobic incubator (10% H_2_, 5% CO_2_, and 85% N_2_). The BHI supplemented with 1% sucrose (wt/vol) (named BHIS) was used for the biofilm assay of S. mutans. Escherichia coli was inoculated in Luria-Bertani medium (1% tryptone, 0.5% yeast extract, 1% NaCl) under aerobic conditions.

### Construction of *ahrC* markerless in-frame deletion mutant, complementation of the Δ*ahrC* mutation, and overexpression of *ahrC*.

The in-frame deletion mutant (Δ*ahrC* mutant) was constructed using a two-step transformation procedure (51). The overexpression strain (UA159/pDL278*-ahrC*) and the complement strain (Δ*ahrC*/pDL278*-ahrC*) were constructed by transferring exogenous plasmids carrying an *ldh* promoter and *ahrC* gene. The purified and digested PCR product was cloned into E. coli-Streptococcus shuttle vector pDL278 linearized before. The obtained plasmid, pDL278-*ahrC*, was directly transformed into the S. mutans UA159 strain and S. mutans Δ*ahrC* strain, and the transformants were selected using BHI agar plates containing spectinomycin (1 mg/mL). All of the PCR products and mutants were confirmed by PCR and DNA sequencing. All primers used are listed in Table S2.

### Planktonic growth assay.

Overnight cultures of S. mutans strains were subcultured into fresh BHI, grown until mid-exponential phase (optical density at 600 nm [OD_600_] = 0.5), and diluted (1:100) using fresh BHI broth. These planktonic bacteria were cultured in 96-well flat-bottom polystyrene microtiter plates (Corning, NY) and grown at 37°C after being covered with sterile mineral oil as previously described (52). Medium alone (without bacteria) was maintained as a blank. The optical density was measured at 600 nm (OD_600_) every hour using a Multiskan Spectrum (Multiskan Go; Thermo, United States).

### Quantification of arginine concentration.

LC-MS was used to quantify arginine content in S. mutans strains (53, 54). The S. mutans strains were cultured overnight and diluted (1:100) with BHI. Then 30 mL of subculture bacteria (OD_600_ = 0.5) was centrifuged at 4,500 × *g* at 4°C for 5 min. After removing the supernatant, the sediment was suspended in 250 μL of the extraction buffer (methanol/acetonitrile/water = 40:40:20 and 0.1 N formic acid) and stored at −20°C for 30 min to lyse the bacterial cells. The thallus lysate was centrifuged at 15,000 × *g* at 4°C for 10 min, and the supernatant was stored as a primary extract at −20°C. The cell fragments were suspended in 125 μL of the extraction buffer, stored at −20°C for 20 min, and centrifuged at 4°C at 15,000 × *g* for 10 min to obtain the secondary extraction supernatant, which was mixed with the primary extraction solution. The mixtures were centrifuged at 15,000 × *g* for 10 min to remove any bacterial debris.

A 100-μL sample of the supernatant was transferred into a fresh tube. The liquid chromatography-tandem mass spectrometry (LC-MS/MS) system consisted of Agilent LC1290-QQQ-6470 (Agilent Technologies, Santa Clara, CA) coupled with the Agilent 1290 Infinity II LC system (Agilent Technologies). The analytes were separated on a Kinetex HILIC column (100 × 2.6 mm inside diameter [i.d.], 2.1 μm; Phenomenex, Torrence, CA) with a SecurityGuard cartridge kit (Phenomenex). Chromatographic separations were performed using a binary gradient mobile phase composed of mobile phase A (0.1% formic acid in distilled water) and mobile phase B (acetonitrile) and programmed as follows: initial condition of 20% of A and 80% of B for 2 min, changing to 70% of A and 30% B for 1.5 min, and changing to 80% of A and 20% of B, followed by maintaining for 1 min. The flow rate was set at 0.45 mL/min for a total run time of 4.5 min, with a column oven temperature of 35°C.

The ESI source was operated in the positive mode, and the mass spectrometer was operated in the multiple reaction monitoring (MRM) mode with a dwell time of 100 ms per MRM channel. Gas temperature, gas flow rate, and nebulizer gas pressure were set at 150°C, 12 L/min, and 30 lb/in^2^, respectively. The selected precursor/product ion pairs were *m/z* 175.1 → 70.1 for arginine.

Standard curves were drawn using different concentrations of l-arginine standards (Solarbio, Beijing, China). When determining the content of the components in a sample, a chromatogram was created using the same chromatographic conditions as the standard curve, and the concentration of the sample components injected into the column was found on the standard curve based on peak area and peak height.

### Crystal violet staining.

The biofilm formation of S. mutans strains was measured by crystal violet staining as previously described (55). Overnight cultures of S. mutans strains were subcultured into fresh BHI, grown until mid-exponential phase (OD_600_ = 0.5), and diluted (1:100) with BHIS. The bacterial dilutions were transferred into 24-well polystyrene microtiter plates (Corning, NY) (1 mL/well) and incubated anaerobically for 24 h at 37°C. After incubation, unattached cells and the medium were gently removed by phosphate-buffered saline (PBS). The biofilms were fixed using 200 μL of 4% paraformaldehyde for 15 min, and then the wells were dried at 37°C. Then, the biofilms were stained with 0.1% crystal violet for 5 min at room temperature. Subsequently, the wells were rinsed with PBS twice, and 200 μL of 33% acetic acid was added to the wells to solubilize the dye under gentle shaking for 30 min. Finally, the acetic acid was transferred to a new plate, and the absorbance was recorded at 570 nm (BioTek).

### Structural imaging of biofilm.

Overnight cultures of S. mutans strains were subcultured into fresh BHI, grown until mid-exponential phase (OD_600_ = 0.5), and diluted (1:100) with BHIS as described above and inoculated anaerobically on glass coverslips for 24 h at 37°C. After incubation, the biofilm was fixed with 2.5% (wt/vol) glutaraldehyde solution for 6 h at 4°C, rinsed with sterile PBS, dehydrated serially (30%, 40%, 50%, 60%, 70%, 80%, 90%, 96%, and 100%) in ethanol and sputter-coated with gold. The sample was examined under a scanning electron microscope (Inspect F50; FEI, USA) at ×1,000, ×5,000, and ×20,000 magnifications.

As described above, S. mutans biofilm was grown on a glass coverslip for 6 h and 24 h to analyze biofilm architecture. Alexa Fluor 647 dextran conjugate (Molecular Probes, Invitrogen, Carlsbad, CA, USA) mixed with BHIS at a final concentration of 1 μM was used to label EPS glucans produced by glucosyltransferases (Gtfs). After incubation, the coverslip was washed twice with double-distilled water to remove planktonic and loosely bound cells. Subsequently, S. mutans was stained with 2.5 μM SYTO 9 (Molecular Probes, Invitrogen, Carlsbad, CA, USA) for 15 min (56). The images were captured with a Nikon confocal laser scanning microscope (CLSM) (Nikon, N-SIM) with a ×60 oil immersion objective lens. The image collection gates were set to 495 to 515 nm for SYTO 9 and 655 to 690 nm for Alexa 647, and each biofilm was scanned at five randomly selected areas. The above results were analyzed for bacteria content, EPS content, biofilm biomass, and biofilm thickness using COMSTAT and ImageJ software, and each set of samples contained at least three replicates.

### RNA sequencing.

Overnight cultures of S. mutans were subcultured into fresh BHI and grown until the mid-exponential phase (OD_600_ = 0.5) under anaerobic conditions. This culture of UA159 and the Δ*ahrC* strain was centrifuged at 10,000 rpm for 2 min at 4°C, and the cell pellet was snap-frozen in liquid nitrogen until needed. The mid-exponential cells of UA159/pDL278 and UA159/pDL278-*ahrC* were then diluted 1:100 in fresh BHI medium supplemented with 1% (wt/vol) sucrose and incubated anaerobically for 6 h at 37°C. Then the culture of UA159/pDL278 and UA159/pDL278-*ahrC* was centrifuged at 10,000 rpm for 2 min at 4°C, and the cell pellet was snap-frozen in liquid nitrogen until needed.

Total RNA was extracted using the mirVana microRNA (miRNA) isolation kit (Ambion, Austin, TX, USA) following the manufacturer’s protocol. RNA integrity was evaluated using the Agilent 2100 Bioanalyzer (Agilent Technologies, Santa Clara, CA, USA). The samples with an RNA integrity number (RIN) of ≥7 were subjected to the subsequent analysis. The libraries were constructed using TruSeq Stranded Total RNA with Ribo-Zero Gold (Illumina, USA) according to the manufacturer’s instructions. Then, these libraries were sequenced on the Illumina sequencing platform (HiSeq 2500), and 150 bp/125 bp paired-end reads were generated. Raw reads generated during high-throughput sequencing were fastq format sequences. In order to get high-quality reads that could be used for later analysis, raw reads were filtered by quality. Trimmomatic (57) software was first used for quality control and linker removal, and then low-quality bases and N-bases or low-quality reads were filtered out. Finally, we got high-quality clean reads. Using Rockhooper2 (58) to align clean reads to the reference genome of the experimental species, the sample was assessed by genomic and gene alignment.

We used S. mutans UA159 complete genome sequences (from NCBI) as the database, and the expression abundance of each gene in each sample was identified by sequence similarity comparison. Rockhooper2 was used to obtain the number of reads aligned to the gene in each sample and then to calculate reads per kilobase per million (RPKM) (59). The calculated transcript expression levels were directly used to compare transcript expression differences between different samples. We used the estimateSizeFactors function of the R package of DESeq (60) to standardize counts and the nbinomTest function to calculate the *P* value and foldchange of difference comparison. Data was visualized through GraphPad Prism version 8.0.2 for Windows (GraphPad Software, San Diego, California USA; www.graphpad.com) to obtain a volcano plot. Transcripts with a |log2FoldChange| of >1 and a *P* value of <0.05 were considered as differentially expressed genes (DEGs). The DEGs were further used for GO enrichment analysis and KEGG pathway analysis (https://david.ncifcrf.gov/).

### Cloning, expression, and purification of recombinant AhrC protein.

The open reading frame (ORF) of *ahrC* was amplified from genomic DNA with the primer pair *ahrC*F/*ahrC*R using a high-fidelity PCR system (TaKaRa). The recombinant vector pET*ahrC* was conducted as described above using pET28a digested with NotI and XhoI. E. coli BL21(DE3) cells harboring pET*ahrC* were grown overnight at 37°C (200 rpm), subcultured into 500 mL of Luria-Bertani medium containing 50 μg/mL kanamycin, and grown at 37°C (200 rpm) until an OD_600_ of 0.5 to 0.7, followed by 4 h of culturing at 30°C after adding 800 μg/mL (final concentration) isopropyl-β-d-thiogalactopyranoside (IPTG) to induce the expression of His-*ahrC* protein. Recombinant protein purification was performed as previously described (61). Protein concentration was determined using spectrophotometry at 280 nm.

### Electrophoretic mobility shift assay.

The gene *smu.661* encodes a helix-turn-helix transcriptional regulator, which exhibited a 2-fold increase in the Δ*ahrC* strain. Since the five genes *smu.580* to *smu.584* have overlapping regions, we chose the 442-bp intergenic region between *smu.577* and *smu.580* as DNA substrates in EMSAs, named *ahrC* promoter. DNA fragments, including *smu.661* promoter p1 (173 bp), *smu.661* promoter p2 (132 bp), *smu.663* (*argC*) promoter (134 bp), and *smu.584* (*ahrC*) promoter (p1, 183 bp; p2, 183 bp; p3, 133 bp), were selected for this assay. The 37-bp fragment *argC* p1 containing the palindromic sequence of the *argC* promoter and the fragment with the palindromic sequence mutated in *argC* p1mut were labeled with FAM (5-carboxyfluorescein) as previously described (62). The fluorescent strand of *argC* p1 and p1mut was annealed with the complementary strand by heating to 95°C followed by slow cooling to room temperature to form a duplex DNA. An increasing concentration of purified His-tagged AhrC was incubated with DNA fragments in binding buffer (20 mM Tris-HCl, 100 mM NaCl, 5% glycerol) for 30 min on ice. The mixture was subjected to nondenaturing polyacrylamide gel electrophoresis in 0.5× TBE buffer at 4°C using the Mini-Protean II system (Bio-Rad, Hercules, CA, USA). For genes not labeled with FAM, the gels were stained with TS-GelRed (nucleic acid gel dyes; Tsingke Biotechnology Co., Ltd.) for 20 min and exposed to a phosphorimager to visualize free DNA and protein-DNA complexes. The gel images were analyzed using Bio-Rad image software.

### DNase I footprinting assay.

DNA fragment *argC*p was amplified using PCR with a special FAM-labeled primer. The DNA fragment and AhrC protein mixture was cleaved at 37°C for 4 min by DNase I (0.1 Kunitz units/μL). The reaction was stopped using phenol-chloroform, and the DNA was precipitated using sodium acetate and ethanol. The DNA fragment precipitate was analyzed on an Applied Biosystems 3730XL DNA analyzer (Tsingke Company, Chengdu). Electropherograms were analyzed and aligned using GENEMAPPER software (Thermo Fisher Scientific, United States). The AhrC binding sequences inferred by MEME (https://meme-suite.org/meme/tools/meme) were visualized using WebLogo to find a conserved sequence (32, 33).

### β-Galactosidase activity analysis.

β-Galactosidase activity was analyzed as previously described (63). The selected promoter sequences were cloned into pDL278 that had already been inserted with *lacZ*. The recombinant plasmids were transformed into UA159 and the Δ*ahrC* strain to obtain the corresponding recombinant strains. All of the strains were grown in BHI agar plates at 37°C for 48 h. A single colony was picked and cultured overnight in liquid BHI and then subcultured into fresh BHI to an OD_600_ of 0.5. After centrifugation, the cell pellets were resuspended with Z buffer (60 mmol/L Na_2_HPO_4_·7H_2_O, 40 mmol/L NaH_2_PO_4_·H_2_O, 10 mmol/L KCl, 1 mmol/L MgSO_4_·7H_2_O, and 50 mmol/L 2-hydroxy-1-ethanethiol, pH = 7.0). The absorbance of the suspension was recorded at 600 nm. Then, chloroform, SDS (sodium dodecyl sulfate), ONPG (2-nitrophenyl β-d-galactopyranoside), and Na_2_CO_3_ were added to the cell suspension, and the absorbance was recorded at 420 and 550 nm. Next, another cell suspension was plated on a BHI-X-gal (5-bromo-4-chloro-3-indolyl-β-d-galactopyranoside) agar plate, and the plate was incubated for 48 h at 37°C for imaging.

### Statistical analysis.

All of the experiments were performed at least in triplicate and reproduced three separate times. Statistical analyses were performed using SPSS 21.0 for Windows (SPSS, Inc.) and Prism 9.0 (GraphPad Software Inc., San Diego, CA, USA) with one-way analysis of variance (ANOVA) to compare the means of all the groups, followed by a two-tailed Student's *t* test to compare the means of two groups. Kruskal-Wallis test was used for nonparametric testing. A two-tailed *P* value of <0.05 was considered statistically significant.

### Data availability.

The raw data from the transcriptomic sequencing analysis of S. mutans UA159, the S. mutans Δ*ahrC* strain, S. mutans UA159/pDL278, and S. mutans UA159/pDL278-*ahrC* have been deposited in the NCBI Sequence Read Archive (SRA) database under BioProject accession number PRJNA812757.
