# Supplementary material for: AhrC Negatively Regulates Streptococcus mutans Arginine Biosynthesis
Source: Microbiol Spectr. 2022 Aug 8;10(4):e00721-22. doi: 10.1128/spectrum.00721-22 (PMC9430756; doi:10.1128/spectrum.00721-22)
Supplement: Supplemental file 1 — Supplemental material. Download spectrum.00721-22-s0001.pdf, PDF file, 0.9 MB [file spectrum.00721-22-s0001.pdf]

SUPPLEMENTAL MATERIALS

FIGURES AND FIGURE LEGENDS

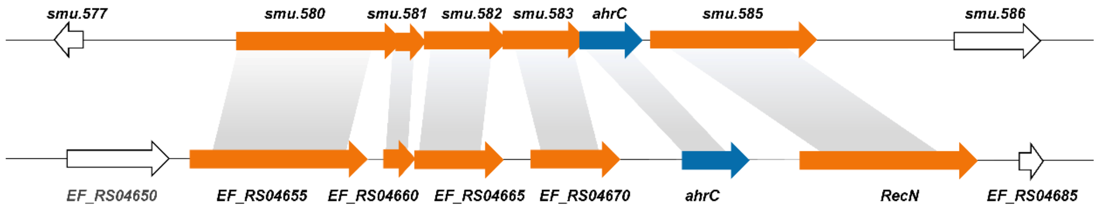

FIG S1 Schematic representation of the genomic architecture of *ahrC* loci in *S. mutans* UA159 and *E. faecalis* OG1RF. The gene *ahrC* is shown in blue, and the homolog genes are shown in orange. According to NCBI annotation, *smu.580* and *EF_RS04655* encode the exodeoxyribonuclease VII large subunit, *smu.581* and *ES_RS04660* encode the exodeoxyribonuclease VII small subunit, *smu.582* and *EF_RS04665* encode the polyprenyl synthetase family protein, *smu.583* and *EF_RS04670* encode the TlyA family rRNA(cytidine-2'-O-)-methyltransferase, *ahrC* encodes the ArgR family transcriptional regulator, and *smu.585* and *recN* encode the DNA repair protein RecN.

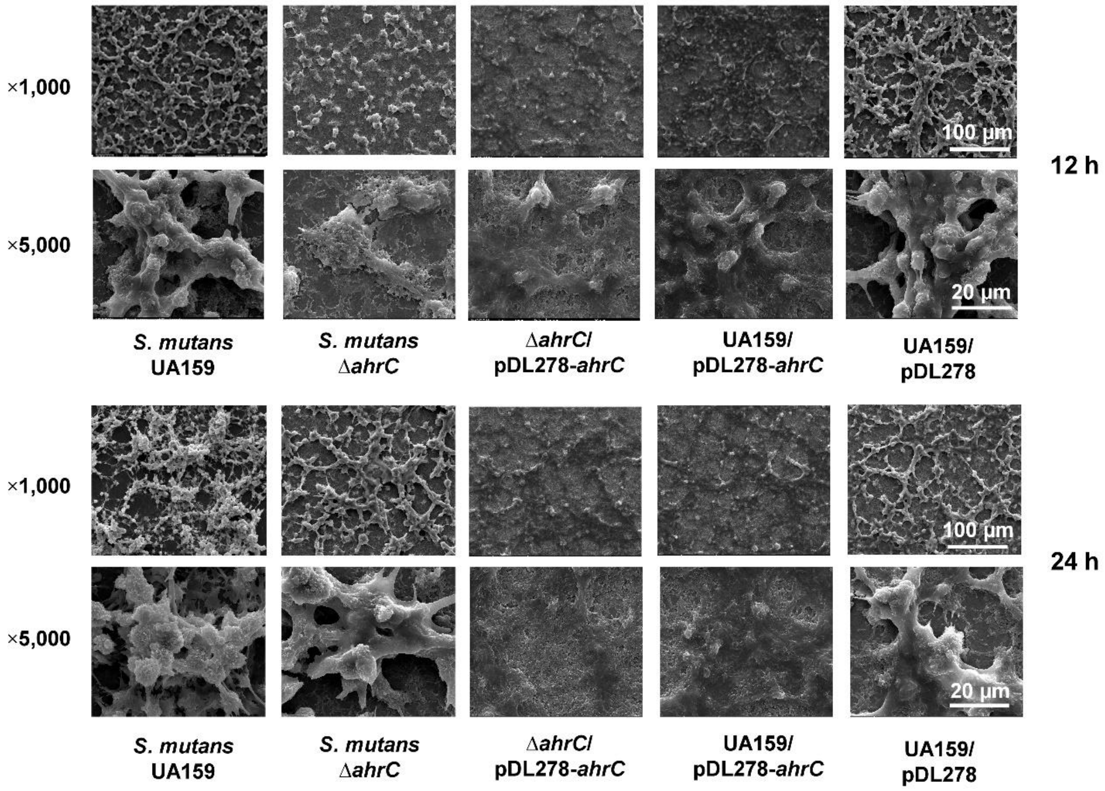

FIG S2 The scanning electron microscope (SEM) images showing the 12-h and 24-h biofilms of *S. mutans* strains. Images were taken at ×1,000 and ×5,000 magnifications.

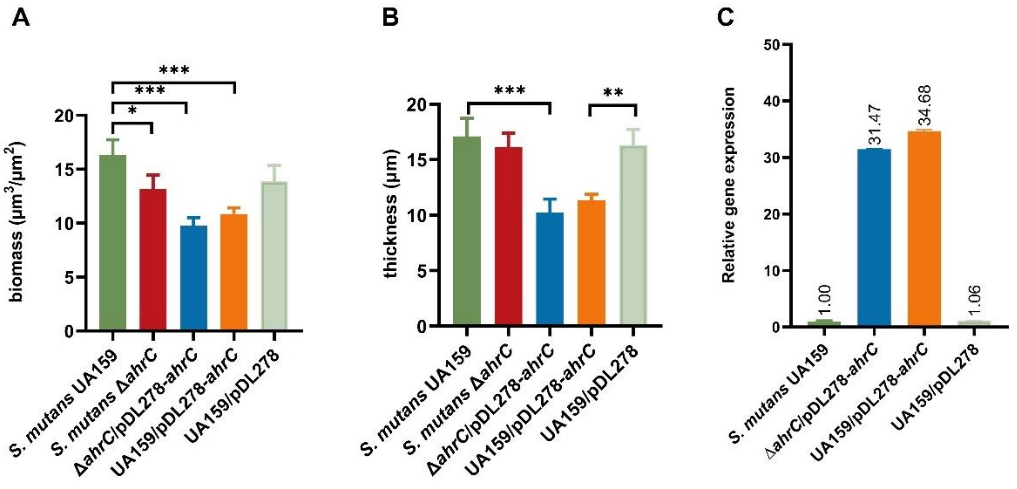

FIG S3 The biomass (A) and thickness (B) of 6-h *S. mutans* biofilms. (C) The relative gene expression of *ahrC* in *S. mutans* strains. The results were the mean values of three samples, expressed as mean ± standard deviation.

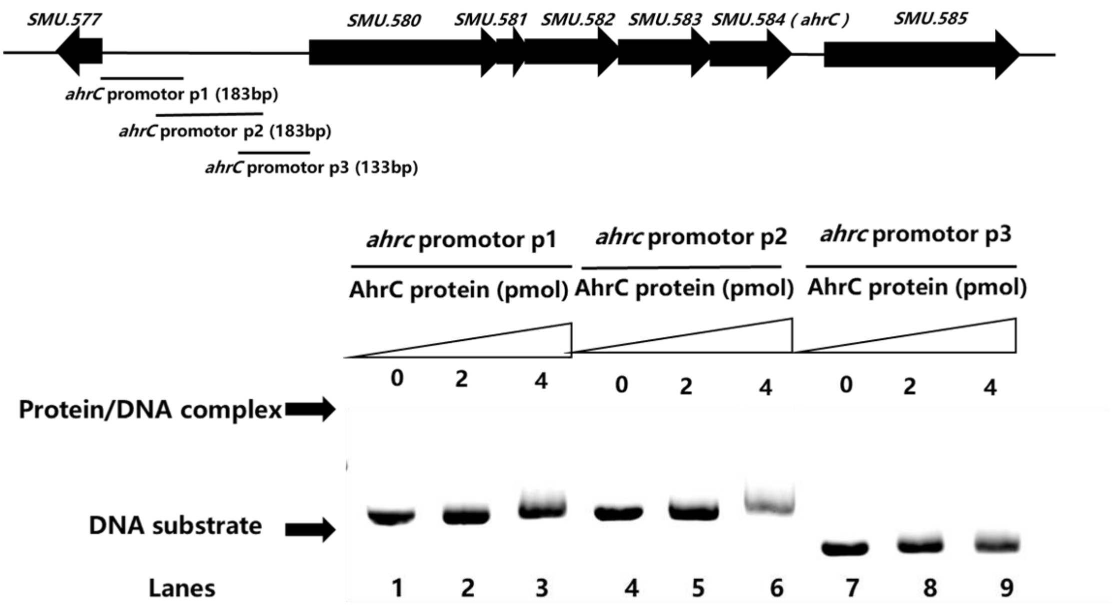

FIG S4 The result of electrophoretic mobility shift assay (EMSA) showing AhrC protein cannot bind to the *ahrC* promoter.

TABLES

Table S1. Bacterial strains and plasmids used in this study

Strains or plasmid	Description	Source
S. mutans		
UA159	Wild type strain	ATCC 700610
Δ*ahrC*	UA159Δ*ahrC*; *Em*^{s}; *p*-Cl-Phe^{r}	This study
Δ*ahrC*/pDL278-*ahrC*	Δ*ahrC*/pDL278-*ldh-ahrC*; Spe^{r}	This study
UA159/pDL278-*ahrC*	UA159/pDL278-*ldh-ahrC*; Spe^{r}	This study
UA159/pDL278	UA159/pDL278; Spe^{r}	This study
ΔS1	UA159Δ*ahrC*/pDL278-*lacZ*; Spe^{r}	This study
S1	UA159/pDL278-*lacZ*; Spe^{r}	This study
ΔS2	UA159Δ*ahrC*/pDL278-*argC*p-*lacZ*; Spe^{r}	This study
S2	UA159/pDL278-*argC*p-*lacZ*; Spe^{r}	This study
ΔS3	UA159Δ*ahrC*/pDL278-*ldh*p-*lacZ*; Spe^{r}	This study
S3	UA159/pDL278-*ldh*p-*lacZ*; Spe^{r}	This study
E. coli		
DH5α	F-, φ 80d*lacZ* ΔM15, Δ(*lacZYA -argF*)U169, *deoR*, *recA1*, *endA1*, *hsdR17* (*rK-*, *mK+*), *phoA*, *supE44*, *λ-*, *thi -1*, *gyrA96*, *relA1*	Laboratory stock
BL21(DE3)	F-, *ompT*, *hsdSB* (rB-mB-), *gal*, *dcm* (DE3)	Novagen
**Plasmids**		
pDL278	*E. coli-Streptococcus* shuttle vector; Spe^{r}	LeBlanc et al., 1992
pDL278-*ahrC*	pDL278 derivative for overexpression mutant of *ahrC* in *S. mutans* UA159 and complementation of *ahrC* in *S. mutans* Δ*ahrC*	This study
pET28a	Kan^{r} expression vector with 6His-tag coding sequence	Novagen
pET*ahrC*	pET derivative for expression 6His-AhrC	This study
pDL278-*lacZ*	pDL278 derivative for β-galactosidase activity analysis in *S. mutans* UA159 and *S. mutans* Δ*ahrC*	This study
pDL278-*argC*p-*lacZ*	pDL278 derivative for β-galactosidase activity analysis in *S. mutans* UA159 and *S. mutans* Δ*ahrC*	This study

pDL278-*ldh*p-*lacZ*	pDL278 derivative for β-galactosidase activity analysis in *S. mutans* UA159 and *S. mutans* Δ*ahrC*	This study

Table S2. Primers used in this study

Primers	Sequence (5' to 3')	Used in
ahrC-upF	CCATGCCTAAGGAAAGAGTG	In-frame deletion construction
ahrC-upR	CTATGAGTGTTATTGTTGCTCGGTCGTTCACGTTTCTTCATTC	In-frame deletion construction
ahrC-dnF	GGTATACTACTGACAGCTTCATCATAAGGAGTGGGTATGC	In-frame deletion construction
ahrC-dnR	GTCTCTAGACGTTTGCTAAC	In-frame deletion construction
ahrC-checkF	GACCCCACAAAAGGATATCG	In-frame deletion construction
ahrC-checkR	CATCAACTGAACCACCATAC	In-frame deletion construction
ahrC-updnF	GAATGAAGAAACGTGAACGAATCATAAGGAGTGGGTATGC	In-frame deletion construction
ahrC-updnR	TCGTTCACGTTTCTTCATTC	In-frame deletion construction
*ldh*F	CGGCCAGTGAATTCGAGCTCAGAGCCCGAGCAACAATAACACT	In-frame deletion construction
ldhR	GTTTCTTCATGTTCTAAACATCTCCTTATAATTT	In-frame deletion construction
ahrC-overF	TGTTTAGAACATGAAGAAACGTGAACGATTAGAA	Complementation and overexpression construction
ahrC-overR	TTGCATGCCTGCAGGTCGACTTAATTGGCCATCCAAGATTTGA	Complementation and overexpression construction
*ahrC*F	TATATAGCGGCCGCTATGAAGAAACGTGAACGATTAG	pET*ahrC* construction
*ahrC*R	CCCCTTCTCGAGTTAATTGGCCATCCAAGATTTGATTTC	pET*ahrC* construction
smu.661-p1F	ACTTTATTTTATTAATATCTTTT	EMSA
smu.661-p1R	CAGCGGTTTCTTCTATTCTCAC	EMSA
smu.661-p2F	TTATTTTACAATTTTACTTTGT	EMSA
smu.661-p2R	CAAATATATCCTCATTTCTC	EMSA
argC-pF	AATAATTTTGTTATCATGAGTT	EMSA
argC-pR	ACTTATTCACTTCCTTTATAAA	EMSA
ahrC-p1F	ATTATGCTTCTCCTATTATTG	EMSA
ahrC-p1R	CCTAACCTTTTCATGGCAATC	EMSA

ahrC-p2F	GTTACTGGTTAGATTGCCATG	EMSA
ahrC-p2R	GGCTCCTTTTCCGTTAATATACG	EMSA
ahrC-p3F	cagctatattaacggaaaagg	EMSA
ahrC-p3R	AAATCTCATCTAAAGACC	EMSA

Table S3. List of genes upregulated >2.0-fold in *S. mutans* Δ*ahrC*

Gene No.	Gene Name	NCBI Annotation	*p*val	*p*adj	Fold Change
smu.179		NAD(P)H-dependent oxidoreductase	3.94E-16	6E-15	2.550491813
smu.190		flavocytochrome c	2.27E-28	5.73E-27	2.789166542
smu.308		SDR family oxidoreductase	1.14E-25	2.63E-24	3.16393653
smu.309		PTS sugar transporter subunit IIA	3.47E-56	1.61E-54	4.446736174
smu.310		transcriptional regulator GutM	4.93E-32	1.41E-30	4.456097683
smu.311		PTS glucitol/sorbitol transporter subunit IIC	3.28E-48	1.26E-46	5.076119496
smu.312		PTS glucitol/sorbitol transporter subunit IIB	8.38E-55	3.63E-53	5.194201221
smu.313		PTS glucitol/sorbitol transporter subunit IIA	9.67E-37	3.12E-35	5.202897486
smu.314		hypothetical protein	1.41E-19	2.5E-18	5.4122531
smu.334	*argG*	argininosuccinate synthase	0	0	39.77668153
smu.335	*argH*	argininosuccinate lyase	0	0	34.10032573
smu.336		ribonuclease P protein component	4.66E-63	2.32E-61	9.520569547
smu.337		membrane protein insertase YidC1	4.8E-89	3.42E-87	7.61676741
smu.338		protein jag	6.1E-111	6.4E-109	6.556629247
smu.339		DUF998 domain-containing protein	2.09E-61	1.02E-59	5.965361626
smu.340		50S ribosomal protein L34	2.21E-30	5.96E-29	3.183237281
smu.402		formate C-acetyltransferase	4.23E-28	1.05E-26	2.395959894
smu.405c		helix-turn-helix transcriptional regulator	5.29E-12	6.28E-11	2.737349847
smu.411c		hypothetical protein	1.76E-09	1.66E-08	2.099013751
smu.503c		hypothetical protein	6.3E-30	1.68E-28	3.990594736
smu.508		Cof-type HAD-IIB family hydrolase	0.004805	0.016869	2.099638459
smu.510c		hypothetical protein	1.4E-19	2.5E-18	3.059689501
smu.563	*argF*	ornithine carbamoyltransferase	0	0	111.9875586
smu.564		DUF1797 family protein	2.55E-97	2.12E-95	9.96698093
smu.567		amino acid ABC transporter permease	7.77E-52	3.3E-50	3.494965697
smu.568		amino acid ABC transporter ATP-binding protein	7.02E-55	3.11E-53	3.606539319
smu.569		ferrous iron transport protein A	1.81E-29	4.75E-28	2.947216589
smu.570		ferrous iron transport protein B	3E-36	9.49E-35	2.794641787
smu.571		FeoB-associated Cys-rich membrane protein	1.49E-16	2.38E-15	3.053587506
smu.574c		antiholin-like protein LrgB	6.12E-40	2.14E-38	5.435212877
smu.575c		CidA/LrgA family protein	9.21E-49	3.6E-47	6.808279162
smu.609		SH3 domain-containing protein	7.5E-49	2.99E-47	8.419835356
smu.640c		PLP-dependent aminotransferase family protein	8.67E-05	0.000452	2.058163208
smu.661		helix-turn-helix transcriptional regulator	6.32E-05	0.000336	2.00722922
smu.663	*argC*	N-acetyl-gamma-glutamyl-phosphate reductase	0	0	752.2387287
smu.664	*argJ*	bifunctional ornithine acetyltransferase/N-acetylglutamate synthase	0	0	783.3371353

smu.665	*argB*	acetylglutamate kinase	0	0	1110.730852
smu.666	*argD*	acetylornithine transaminase	0	0	149.7259952
smu.670	*acnA*	aconitate hydratase AcnA	2.14E-07	1.64E-06	2.353488142
smu.671	*citZ*	citrate synthase	1.37E-07	1.07E-06	2.266664142
smu.672	*icd*	NADP-dependent isocitrate dehydrogenase	4.33E-11	4.75E-10	2.25249763
smu.673		putative ABC transporter permease	5.1E-15	7.59E-14	2.026962987
smu.674		phosphocarrier protein HPr	5.34E-18	9.03E-17	2.129456226
smu.768c		hypothetical protein	1.81E-06	1.26E-05	2.389187968
smu.799c		thioesterase family protein	2.1E-17	3.4E-16	2.797192607
smu.803c		ABC-F family ATP-binding cassette domain-containing protein	4.06E-12	4.97E-11	2.072063524
smu.815		transporter substrate-binding domain-containing protein	0	0	31.98827785
smu.816		aminotransferase	1.1E-130	1.5E-128	7.897249357
smu.817		transporter substrate-binding domain-containing protein	4.82E-87	3.32E-85	5.729397426
smu.840c		hypothetical protein	1.22E-24	2.61E-23	2.444897191
smu.856	*pyrR*	bifunctional pyr operon transcriptional regulator/uracil phosphoribosyltransferase PyrR	2.71E-70	1.5E-68	7.192000394
smu.857		uracil permease	1.9E-125	2.2E-123	8.23034758
smu.858		aspartate carbamoyltransferase catalytic subunit	9.65E-86	6.21E-84	9.241318366
smu.859	*carA*	glutamine-hydrolyzing carbamoyl-phosphate synthase small subunit	2.7E-154	4.4E-152	9.671455023
smu.860	*carB*	carbamoyl-phosphate synthase large subunit	1.3E-163	2.3E-161	9.761107682
smu.871		alpha-galactosidase	1.89E-28	4.82E-27	2.636914568
smu.878		extracellular solute-binding protein	6.16E-27	1.5E-25	2.77085967
smu.879		sugar ABC transporter permease	3.32E-22	6.48E-21	2.822139097
smu.880		carbohydrate ABC transporter permease	2.86E-21	5.43E-20	2.616586836
smu.881	*gtfA*	sucrose phosphorylase	1.81E-28	4.7E-27	2.691597766
smu.882	*ugpC*	sn-glycerol-3-phosphate ABC transporter ATP-binding protein UgpC	3.03E-21	5.7E-20	2.405256257
smu.883		alpha-glucosidase	1.77E-25	4.01E-24	2.489143628
smu.984		CHAP domain-containing protein	1.96E-14	2.83E-13	6.638684222
smu.985		glycoside hydrolase family 1 protein	1.63E-20	3.01E-19	2.889831375
smu.1013c		CitMHS family transporter	1.43E-18	2.46E-17	3.05375137
smu.1067c		ABC transporter permease subunit	6.51E-14	9.02E-13	2.055205368
smu.1068c		ABC transporter ATP-binding protein	5.64E-18	9.46E-17	2.442149939
smu.1069c		DUF3021 family protein	1.45E-22	2.92E-21	2.738456795
smu.1070c		LytTR family transcriptional regulator DNA-binding domain-containing protein	3.09E-35	9.49E-34	3.434863397
smu.1072c		N-acetyltransferase	1.23E-05	7.46E-05	2.053306369
smu.1077		phospho-sugar mutase	6.85E-44	2.53E-42	3.05091102

smu.1088		FAD:protein FMN transferase	7.61E-27	1.81E-25	2.428005073
smu.1089		NAD(P)H-dependent oxidoreductase	9.03E-33	2.61E-31	2.728249715
smu.1090		NAD(P)H-dependent oxidoreductase	3.74E-37	1.24E-35	2.851500192
smu.1217c		transporter substrate-binding domain-containing protein	6.65E-27	1.6E-25	2.733823688
smu.1218		amidase	4.13E-64	2.12E-62	4.487021238
smu.1219c		MmcQ/YjbR family DNA-binding protein	1.61E-50	6.67E-49	4.998031395
smu.1220c		YbhB/YbcL family Raf kinase inhibitor-like protein	8.17E-56	3.71E-54	4.637525417
smu.1221		orotate phosphoribosyltransferase	6.83E-81	4.26E-79	5.156847351
smu.1222	*pyrF*	orotidine-5'-phosphate decarboxylase	4.97E-74	2.92E-72	5.046765613
smu.1223		dihydroorotate dehydrogenase	9.5E-135	1.3E-132	10.38542234
smu.1224		dihydroorotate dehydrogenase electron transfer subunit	1.1E-101	1.1E-99	9.228122515
smu.1402c	*csn2*	type II-A CRISPR-associated protein Csn2	1.88E-18	3.21E-17	2.954527438
smu.1403c	*cas2*	CRISPR-associated endonuclease Cas2	2.58E-15	3.87E-14	2.995641902
smu.1404c	*cas1*	type II CRISPR-associated endonuclease Cas1	4.04E-25	9.06E-24	2.829037101
smu.1405c	*cas9*	type II CRISPR RNA-guided endonuclease Cas9	1.63E-35	5.1E-34	2.9451704
smu.1421		2-oxo acid dehydrogenase subunit E2	1.25E-09	1.19E-08	3.413624097
smu.1422		alpha-ketoacid dehydrogenase subunit beta	2.88E-10	2.9E-09	3.123083647
smu.1423		thiamine pyrophosphate-dependent dehydrogenase E1 component subunit alpha	0.000633	0.002717	3.324343936
smu.1424	*lpdA*	dihydrolipoyl dehydrogenase	9.43E-11	1.02E-09	3.173489991
smu.1491	*lacE*	PTS transporter subunit EIIC	5.08E-09	4.67E-08	2.161362681
smu.1492	*lacF*	PTS lactose/cellobiose transporter subunit IIA	0.00466	0.016397	2.116438351
smu.1493	*lacD*	tagatose-bisphosphate aldolase	0.0002	0.000962	2.120493267
smu.1494	*lacC*	tagatose-6-phosphate kinase	1E-07	8.02E-07	2.8057608
smu.1495	*lacB*	galactose-6-phosphate isomerase subunit LacB	0.002917	0.010758	3.036939984
smu.1496	*lacA*	galactose-6-phosphate isomerase subunit LacA	0.000429	0.001921	3.65497423
smu.1535		glycogen/starch/alpha-glucan phosphorylase	2.13E-21	4.08E-20	2.176582656
smu.1536	*glgA*	glycogen synthase GlgA	9.7E-37	3.12E-35	2.878486094
smu.1537	*glgD*	glucose-1-phosphate adenylyltransferase subunit GlgD	2.55E-42	9.08E-41	3.227890659
smu.1538	*glgC*	glucose-1-phosphate adenylyltransferase	1.12E-39	3.86E-38	3.591978262
smu.1539	*glgB*	1%2C4-alpha-glucan branching protein GlgB	3.03E-11	3.34E-10	3.054993594
smu.1564	*glgP*	glycogen/starch/alpha-glucan phosphorylase	4.95E-26	1.16E-24	2.372343557

smu.1565	*malQ*	4-alpha-glucanotransferase	2.54E-23	5.22E-22	2.279363771
smu.1568	*malX*	extracellular solute-binding protein	1.9E-14	2.76E-13	2.823366943
smu.1569	*malF*	sugar ABC transporter permease	2.23E-22	4.45E-21	2.67861185
smu.1570	*malG*	sugar ABC transporter permease	2.44E-16	3.78E-15	2.929490153
smu.1571	*ugpC*	sn-glycerol-3-phosphate ABC transporter ATP-binding protein UgpC	1.02E-24	2.24E-23	3.116495551
smu.1596	*celB*、*ptcC*	PTS cellobiose transporter subunit IIC	2.58E-24	5.48E-23	219.1534571
smu.1597c		hypothetical protein	2.31E-45	8.69E-44	322.7828984
smu.1598	*ptcA*	PTS cellobiose transporter subunit IIA	8.2E-103	8.2E-101	234.4408436
smu.1599	*celR*	BglG family transcription antiterminator	1.78E-70	1.02E-68	139.4929301
smu.1600	*ptcB*	PTS cellobiose transporter subunit IIB	0	0	118.9742203
smu.1601	*bgl*	6-phospho-beta-glucosidase	2.39E-31	6.62E-30	84.76079073
smu.1843	*scrB*	sucrose-6-phosphate hydrolase	1.98E-16	3.1E-15	2.182876444
smu.1844	*scrR*	LacI family DNA-binding transcriptional regulator	7.5E-18	1.25E-16	2.365525345
SMU_RS09315		hypothetical protein	7.26E-20	1.32E-18	5.03004718
smu.2037	*treA*	alpha%2Calpha-phosphotrehalase	3.88E-77	2.35E-75	4.510115195
smu.2038	*pttB*	PTS system trehalose-specific EIIBC component	1.98E-43	7.18E-42	3.83428137
smu.2046c		endonuclease/exonuclease/phosphatase family protein	1.47E-34	4.44E-33	2.761416969
smu.2047	*ptsG*	PTS transporter subunit EIIC	1.15E-25	2.63E-24	2.31048724
smu.2127		NAD-dependent succinate-semialdehyde dehydrogenase	2.85E-34	8.5E-33	2.706548857

Table S4. List of genes downregulated >2.0-fold in *S. mutans* Δ*ahrC*

Gene No.	Gene Name	NCBI Annotation	*p*val	*p*adj	Fold Change
smu.61		helix-turn-helix transcriptional regulator	1.78E-12	2.26E-11	2.178181644
smu.113		1-phosphofructokinase	0.00013515	0.000675752	2.006670709
smu.127		pyruvate dehydrogenase	9.29E-12	1.07E-10	3.120012472
smu.128		alpha-ketoacid dehydrogenase subunit beta	2.55E-14	3.60E-13	3.488730038
smu.129		dihydrolipoamide acetyltransferase	1.11E-15	1.68E-14	3.198659776
smu.130		dihydrolipoyl dehydrogenase	2.34E-14	3.34E-13	2.949576688
smu.131		lipoate--protein ligase	1.17E-10	1.24E-09	2.788932437
smu.132		amidohydrolase	3.04E-09	2.83E-08	2.676562563
smu.137		NAD-dependent malic enzyme	2.57E-89	1.90E-87	7.124522926
smu.138		AEC family transporter	4.75E-91	3.64E-89	8.468958495
smu.139		oxalate decarboxylase family bicupin	2.26E-98	2.05E-96	8.574470684
smu.140		NAD(P)/FAD-dependent oxidoreductase	5.64E-98	4.90E-96	7.963905361
smu.141		DUF1275 domain-containing protein	1.30E-91	1.04E-89	8.636003414
smu.151		hypothetical protein	0.034607905	0.089202547	3.917919473
smu.152		hypothetical protein	0.010726509	0.033384377	3.46037506
SMU_RS00765		helix-turn-helix transcriptional regulator	0.026469705	0.071651374	3.042997379
smu.263		APC family permease	0.002404456	0.009033689	2.209951285
smu.364		type I glutamate--ammonia ligase	6.78E-13	8.90E-12	2.205334696
smu.396		aquaporin family protein	4.13E-19	7.16E-18	2.191038309
smu.423		ComC/BlpC family leader-containing pheromone/bacteriocin	0.025982352	0.070619608	3.035361826
smu.463		thioredoxin-disulfide reductase	2.25E-05	0.000128859	2.036926271
smu.524		ATP-binding cassette domain-containing protein	1.33E-19	2.39E-18	2.486890816
smu.525		ABC transporter ATP-binding protein	3.65E-19	6.39E-18	2.42533454
SMU_RS02705		IS3-like element ISSmu1 family transposase	5.12E-12	6.12E-11	2.645990762
smu.584		ArgR family transcriptional regulator	3.05E-126	3.80E-124	252.6966629
smu.600c		folate family ECF transporter S component	3.33E-16	5.10E-15	2.260888887
smu.602		bile acid:sodium symporter family protein	1.70E-05	0.000100579	2.764444086
smu.629		superoxide dismutase	7.89E-09	7.02E-08	2.788928407
smu.667	*nrdF*	class 1b ribonucleoside-diphosphate reductase subunit beta	3.16E-22	6.24E-21	2.313015555
smu.668c	*nrdE*	class 1b ribonucleoside-diphosphate reductase subunit alpha	3.96E-13	5.27E-12	2.244254161

smu.669c		redoxin NrdH	6.73E-10	6.55E-09	2.143600029
smu.764	*ahpC*	peroxiredoxin	9.70E-05	0.000496009	2.341626226
smu.765	*ahpF*	alkyl hydroperoxide reductase subunit F	9.21E-06	5.67E-05	2.723906145
SMU_RS03570		IS3-like element ISSmu1 family transposase	4.15E-12	5.05E-11	2.653255057
smu.819		large conductance mechanosensitive channel protein MscL	6.89E-10	6.67E-09	2.215716873
smu.870		DeoR/GlpR transcriptional regulator	1.78E-10	1.86E-09	2.151421038
smu.871	*pfkB*	1-phosphofructokinase	3.60E-10	3.59E-09	2.110101106
smu.872		PTS sugar transporter subunit IIA	5.82E-09	5.32E-08	2.966291383
smu.873	*metE*	5-methyltetrahydropteroyltriglutamate--homocysteine S-methyltransferase	0.000228126	0.001081025	2.778729685
smu.910		glucosyltransferase-S	8.80E-31	2.40E-29	2.690274335
smu.924		thiol peroxidase	0.005254963	0.018104289	2.531086042
smu.929c		DUF1304 family protein	2.00E-06	1.37E-05	3.274618488
smu.992		hypothetical protein	2.40E-23	4.98E-22	2.75109147
smu.993		ribosome biogenesis GTPase YlqF	7.58E-12	8.79E-11	2.035523258
smu.1175		sodium:alanine symporter family protein	1.59E-07	1.24E-06	3.5008595
smu.1185		PTS mannitol transporter subunit IICBA	4.78E-34	1.40E-32	3.311751958
smu.1243		low temperature requirement protein A	6.19E-09	5.61E-08	2.744592919
smu.1296	*yghU*	glutathione-dependent disulfide-bond oxidoreductase	1.33E-05	7.98E-05	2.817053251
smu.1297		bifunctional oligoribonuclease/PAP phosphatase NrnA	2.55E-11	2.82E-10	2.1327506
smu.1337c		mutanobactin A biosynthesis alpha/beta hydrolase MubM	0.001313271	0.005219074	2.082219237
SMU_RS06285		IS3-like element ISSmu1 family transposase	3.52E-12	4.37E-11	2.658098101
SMU_RS06355		hypothetical protein	2.62E-07	1.99E-06	5.594657998
smu.1396	*gbpC*	glucan-binding protein	8.89E-18	1.47E-16	3.822592123
SMU_RS06410		IS3-like element ISSmu1 family transposase	4.49E-12	5.43E-11	2.652022993
smu.1502c		membrane protein	4.75E-08	3.93E-07	2.106677786
smu.1645		SAM-dependent methyltransferase TehB	9.12E-22	1.77E-20	2.597524935
smu.1649		exodeoxyribonuclease III	1.09E-17	1.78E-16	2.303713549
smu.1657c		P-II family nitrogen regulator	0.031058087	0.082182936	2.055365187
smu.1658	*nrgA*	ammonium transporter	0.000277344	0.001293926	2.230120165
smu.1700c		LrgB family protein	3.57E-14	5.01E-13	2.339320366
smu.1702c		phosphatase PAP2 family protein	5.75E-20	1.05E-18	2.389281756
smu.1703c		ECF transporter S component	2.22E-16	3.46E-15	2.357531996
smu.1787c		preprotein translocase subunit YajC	0.000462394	0.002054512	2.514639788
smu.1865		A/G-specific adenine glycosylase	6.92E-13	9.02E-12	2.601560043
smu.1875		ATP-dependent RecD-like DNA helicase	7.03E-21	1.31E-19	3.628908853

smu.1876		hypothetical protein	1.50E-27	3.70E-26	3.729550223
smu.1877	*ptnA*	PTS mannose transporter subunit IIAB	1.25E-31	3.52E-30	185.0957241
smu.1878		PTS mannose/fructose/sorbose transporter subunit IIC	4.08E-86	2.71E-84	231.4607636
smu.1879		PTS mannose/fructose/sorbose transporter family subunit IID	1.17E-24	2.53E-23	14.62244139
SMU_RS08640		IS3-like element ISSmu1 family transposase	4.90E-12	5.89E-11	2.648005326
smu.1895c		ComC/BlpC family leader-containing pheromone/bacteriocin	2.36E-09	2.22E-08	2.35774035
smu.1902c		hypothetical protein	2.13E-17	3.43E-16	3.107582146
smu.1904c		thiol reductase thioredoxin	2.10E-08	1.78E-07	3.381457732
smu.1905c		ComC/BlpC family leader-containing pheromone/bacteriocin	4.15E-07	3.07E-06	3.591881858
smu.1906c		hypothetical protein	1.99E-12	2.51E-11	3.283866642
smu.1908c		hypothetical protein	1.34E-06	9.47E-06	3.684581133
smu.1909c		hypothetical protein	6.60E-09	5.90E-08	3.638947118
smu.1910c		hypothetical protein	3.01E-07	2.27E-06	3.449320229
smu.1913c		hypothetical protein	4.96E-05	0.000269575	3.687256147
smu.1914c		ComC/BlpC family leader-containing pheromone/bacteriocin	1.07E-10	1.15E-09	3.147326144
smu.1916	*comD*	GHKL domain-containing protein	7.69E-11	8.33E-10	2.419150016
smu.1917	*comE*	response regulator transcription factor	2.22E-14	3.18E-13	2.003686707
smu.1980c		hypothetical protein	0.000658505	0.00280709	2.229207401
smu.1981c		ComGF family competence protein	0.000142624	0.000709562	2.372161795
smu.1983	*comYD*	type II secretion system protein	0.005399926	0.018541916	2.090067169
smu.1984	*comYC*	prepilin-type N-terminal cleavage/methylation domain-containing protein	8.51E-05	0.000446881	2.255351028
smu.1987	*comYA*	Flp pilus assembly complex ATPase component TadA	0.000193539	0.00093716	2.445153273
smu.1988c		DUF1033 family protein	1.89E-05	0.000110147	2.003060331
smu.2133c		YhgE/Pip domain-containing protein	5.01E-06	3.22E-05	2.123544603
smu.2141		tRNA uridine-5-carboxymethylaminomethyl(34) synthesis enzyme MnmG	5.53E-10	5.46E-09	2.024474154
smu.2146c		transglycosylase SLT domain-containing protein	7.33E-23	1.49E-21	2.758231401

Table S5. List of genes upregulated >2.0-fold in *S. mutans* UA159/pDL278-*ahrC*

Gene No.	Gene Name	NCBI Annotation	*p*val	*p*adj	fold change
smu.137		NAD-dependent malic enzyme	5.37E-70	2.63E-67	7.01262413
smu.138		AEC family transporter	3.27E-76	2.14E-73	9.08824469
smu.139		oxalate decarboxylase family bicupin	2.17E-65	8.53E-63	7.12034235
smu.140		NAD(P)/FAD-dependent oxidoreductase	3.05E-65	9.98E-63	6.80789756
smu.141		DUF1275 domain-containing protein	5.14E-55	1.12E-52	6.27504006
smu.191c		site-specific integrase	0.00192032	0.021784805	3.0476488
smu.193c		DUF3173 domain-containing protein	0.043047856	0.174144112	4.34143076
smu.194c		DUF3850 domain-containing protein	0.009608503	0.067328156	6.89440154
smu.195c		hypothetical protein	0.013557887	0.086365503	4.47583552
smu.196c		CHAP domain-containing protein	4.92E-06	0.0001509	2.85610408
smu.197c		hypothetical protein	9.33E-17	9.64E-15	4.04046091
smu.198c		ATP-binding protein	2.92E-21	3.58E-19	4.82968359
smu.199c		conjugal transfer protein	9.80E-10	5.34E-08	17.0996519
smu.200c		hypothetical protein	0.002947506	0.029060336	11.8421478
smu.201c		conjugal transfer protein	9.32E-14	7.32E-12	7.03959375
smu.202c		hypothetical protein	1.23E-07	5.34E-06	4.91933202
smu.205c		hypothetical protein	0.008930975	0.064383398	2.83516985
smu.207c		replication initiation factor domain-containing protein	8.82E-12	5.97E-10	4.03592197
smu.208c		transposase	5.45E-15	4.86E-13	6.33433725
smu.209c		DUF961 family protein	6.17E-08	2.88E-06	4.21278334
smu.210c		hypothetical protein	2.19E-12	1.59E-10	7.17548921
smu.214c		hypothetical protein	0.020332678	0.105257822	3.12008372
smu.526c		MerR family transcriptional regulator	1.29E-05	0.000356136	2.02834897
smu.584		ArgR family transcriptional regulator	7.33E-215	1.44E-211	44.1191578
smu.753		PspC domain-containing protein	2.76E-10	1.64E-08	2.45297121
smu.940c		hemolysin III family protein	1.88E-56	4.60E-54	5.32087073
smu.941c		DUF1836 domain-containing protein	1.00E-39	1.64E-37	4.85259455
SMU_RS06070		hypothetical protein	2.44E-05	0.000605422	2.1844479
smu.1628		DUF3397 family protein	0.038429097	0.161579677	2.06740161
smu.1764c		CRISPR-associated helicase/endonuclease Cas3	3.79E-14	3.10E-12	2.2024906
SMU_RS09515		hypothetical protein	5.38E-10	3.02E-08	2.45935342
smu.2083c		hypothetical protein	1.37E-07	5.74E-06	2.05583209
smu.2084c		transcriptional regulator Spx	1.36E-11	8.92E-10	2.90300718
smu.2094c		Crp/Fnr family transcriptional regulator	3.04E-05	0.000736052	2.1826041

Table S6. List of genes downregulated >2.0-fold in *S. mutans* UA159/pDL278-*ahrC*

Gene No.	Gene Name	NCBI Annotation	pval	padj	fold change
smu.49		ATP-grasp domain-containing protein	0.000472155	0.007294234	0.490455617
smu.50	*purE*	5-(carboxyamino)imidazole ribonucleotide mutase	0.000328391	0.005506865	0.44180035
smu.51	*purK*	5-(carboxyamino)imidazole ribonucleotide synthase	0.005950688	0.048331429	0.371313071
smu.52		hypothetical protein	0.01439516	0.088846407	0.487703848
smu.53		hypothetical protein	0.014410603	0.088846407	0.399215894
smu.54		aspartate/glutamate racemase family protein	8.18E-06	0.000243213	0.442452809
smu.t19		tRNA-Leu	0.002084534	0.022976719	0.476110265
smu.113	*pfkB*	1-phosphofructokinase	0.012040278	0.079538808	0.275531238
smu.114		PTS transporter subunit EIIC	0.040044414	0.164710986	0.460954042
smu.150		hypothetical protein	2.07E-07	8.43E-06	0.08223207
smu.151		hypothetical protein	7.73E-30	1.01E-27	0.114631161
smu.152		hypothetical protein	6.62E-12	4.64E-10	0.16361173
		helix-turn-helix transcriptional regulator	4.18E-60	1.17E-57	0.115088983
smu.299c		garvicin Q family class II bacteriocin	0.003510737	0.032800311	0.268546676
SMU_RS01520	*ffs*	signal recognition particle sRNA small type	0.036794816	0.157741108	0.382531326
smu.423		ComC/BlpC family leader-containing pheromone/bacteriocin	8.01E-34	1.12E-31	0.135265564
smu.424		CopY/TcrY family copper transport repressor	0.009089938	0.064852577	0.482132933
smu.426		copper-translocating P-type ATPase	2.63E-06	8.31E-05	0.455447247
smu.494		fructose-6-phosphate aldolase	0.000370552	0.006008455	0.478148792
smu.616		hypothetical protein	0.019518748	0.104064628	0.320373723
smu.664	*argJ*	bifunctional ornithine acetyltransferase/N-acetylglutamate synthase	0.000205585	0.003805259	0.429944222
smu.665	*argB*	acetylglutamate kinase	0.000356776	0.005882312	0.373747725
smu.666	*argD*	acetylornithine transaminase	0.017882879	0.098556764	0.445938519
smu.758c		DUF3270 domain-containing protein	0.000200805	0.003788255	0.472640578
smu.929c		DUF1304 family protein	0.015066358	0.08961398	0.441090987
smu.1072c		N-acetyltransferase	1.78E-05	0.000465187	0.372268971
smu.1091		LPXTG cell wall anchor domain-containing protein	1.14E-17	1.24E-15	0.409962537
smu.1396	*gbpC*	glucan-binding protein	3.55E-16	3.48E-14	0.395987571
smu.1595		carbonic anhydrase family protein	8.38E-13	6.32E-11	0.460310571
smu.1605		DHA2 family efflux MFS transporter permease subunit	0.000869029	0.011758855	0.491449887
smu.1641c		CsbD family protein	0.037095092	0.157741108	0.417628682
smu.1858		30S ribosomal protein S18	0.031689568	0.141980697	0.437755576
smu.1882c		hypothetical protein	0.017757931	0.098143833	0.35881831
smu.1895c		ComC/BlpC family leader-containing pheromone/bacteriocin	0.007811756	0.059176316	0.379207264
smu.1896c		ComC/BlpC family leader-containing pheromone/bacteriocin	0.020290845	0.105257822	0.281907602

smu.1904c	hypothetical protein	3.05E-84	2.99E-81	0.104239879
smu.1905c	thiol reductase thioredoxin	1.59E-20	1.83E-18	0.076086451
smu.1906c	ComC/BlpC family leader-containing pheromone/bacteriocin	3.10E-15	2.89E-13	0.085864852
smu.1909c	hypothetical protein	5.05E-45	9.91E-43	0.09552783
smu.1910c	hypothetical protein	6.76E-40	1.21E-37	0.113515878
smu.1913c	hypothetical protein	8.41E-36	1.27E-33	0.101553628
smu.1914c	hypothetical protein	1.94E-06	6.57E-05	0.053642012
smu.1927	hypothetical protein	4.82E-07	1.78E-05	0.33999381
smu.1928	ComC/BlpC family leader-containing pheromone/bacteriocin	0.000170907	0.003387066	0.484043761
smu.1955	ABC transporter ATP-binding protein	0.003692539	0.034012962	0.37324387
smu.1956c	FtsX-like permease family protein	0.001507041	0.018953938	0.38668222
smu.1957	co-chaperone GroES	0.000668112	0.009430469	0.38926291
smu.1958c	hypothetical protein	0.000763532	0.010549644	0.382779417
smu.1960c	PTS system mannose/fructose/sorbose family transporter subunit IID	0.000258562	0.004449984	0.365269704
smu.1961c	PTS sugar transporter subunit IIC	0.008628842	0.064076661	0.24018487
smu.1902c	PTS sugar transporter subunit IIB	2.08E-14	1.78E-12	0.264553151
smu.1908c	PTS fructose transporter subunit IIA	5.82E-05	0.001283861	0.174829828
